# Selecting a TNT Schedule in Locally Advanced Rectal Cancer: Can We Predict Who Actually Benefits?

**DOI:** 10.3390/cancers15092567

**Published:** 2023-04-30

**Authors:** Carlo Aschele, Robert Glynne-Jones

**Affiliations:** 1 Medical Oncology Unit, Department of Oncology, Ospedale Sant’Andrea, Via Vittorio Veneto 197, 19121 La Spezia, Italy; carlo.aschele@asl5.liguria.it; 2 Radiotherapy Department, Mount Vernon Centre for Cancer Treatment, Mount Vernon Hospital, Rickmansworth Rd., Northwood, London HA6 2RN, UK

**Keywords:** locally advanced rectal cancer, chemoradiation, short-course preoperative radiotherapy, total neoadjuvant therapy, induction chemotherapy, consolidation chemotherapy

## Abstract

**Simple Summary:**

Total neoadjuvant therapy is a strategy developed to improve the efficacy of chemotherapy in locally advanced rectal cancer by anticipating all the chemotherapy before surgery. This improves compliance, early exposure to micrometastatic disease, and local tumor response. In two recent randomized studies, the rates of patients developing distant metastases were indeed reduced, and the proportions of patients showing complete tumor regression at surgery doubled with two different regimens of total neoadjuvant treatment compared to preoperative long-course chemoradiation. Other studies showed that this strategy facilitates rectal preservation with increased rates of clinically complete tumor disappearance without surgery. However, the optimal chemotherapy regimen, radiotherapy schedule, and timing of chemotherapy and radiotherapy are yet to be defined and may not be the same for all risk groups. Additionally, TNT may result in overtreatment for low-risk patients. Indications for this strategy and specific TNT regimens should thus be adapted to different clinical scenarios.

**Abstract:**

Many consider the standard of care for locally advanced rectal cancer (LARC) to be preoperative chemoradiotherapy, radical surgery involving a total mesorectal excision, and post-operative adjuvant chemotherapy based on the pathology of the specimen. The poor impact on distant control is a major limitation of this strategy, with metastasis rates remaining in the 25–35% range and recovery after radical surgery leading to reluctance with prescription and inconsistent patient compliance with adjuvant chemotherapy. A second limitation is the low rate of pathologic complete response (pCR) (around 10–15%) despite multiple efforts to potentiate preoperative chemoradiation regimens, which in turn means it is less effective at achieving non-operative management (NOM). Total neoadjuvant treatment (TNT) is a pragmatic approach to solving these problems by introducing systemic chemotherapy at an early timepoint. Enthusiasm for delivering TNT for patients with LARC is increasing in light of the results of published randomized phase III trials, which show a doubling of the pCR rate and a significant reduction in the risk of subsequent metastases. However, there has been no demonstrated improvement in quality of life or overall survival. A plethora of potential chemotherapy schedules are available around the radiotherapy component, which include preoperative induction or consolidation with a range of options (FOLFOXIRI, FOLFOX, or CAPEOX,) and a varying duration of 6–18 weeks, prior to long course chemoradiation (LCCRT) or consolidation NACT following short-course preoperative radiation therapy (SCPRT) using 5 × 5 Gy or LCCRT using 45–60 Gy, respectively. The need to maintain optimal local control is a further important factor, and preliminary data appear to indicate that the RT schedule remains a crucial issue, especially in more advanced tumors, i.e., mesorectal fascia (MRF) invasion. Thus, there is no consensus as to the optimum combination, sequence, or duration of TNT. The selection of patients most likely to benefit is challenging, as clear-cut criteria to individuate patients benefiting from TNT are lacking. In this narrative review, we examine if there are any necessary or sufficient criteria for the use of TNT. We explore potential selection for the individual and their concerns with a generalized use of this strategy.

## 1. Introduction

Phase III trials of both short-course preoperative radiotherapy (SCPRT) and long course chemoradiation (LCCRT) have achieved significant gains in local control in patients with locally advanced rectal cancer (LARC). National guidelines in the United States have advocated the use of LCCRT for all patients with Stage II and III rectal cancer since 2002, but European guidelines have taken a more risk-adaptive approach [1]. Yet, even with long-term follow-up, fluoropyrimidine based LCCRT alone has shown no impact on the cumulative incidence of distant metastases or improvement in disease-free survival (DFS) [2,3].

Hence, distant metastasis (DM) rather than local recurrence is currently the main cause of treatment failure in LARC. The limitations and challenges of delivering postoperative adjuvant chemotherapy have been addressed [4]. TNT delivers chemotherapy before surgery as a pragmatic solution to delivering timely adjuvant chemotherapy consistently and with appropriate and sufficient doses. Earlier exposure to systemic chemotherapy may be a further advantage of this strategy. In addition, PCR could not be substantially increased in LARC above the 10–15% obtained with fluoropyrimidine-based LCCRT, despite multiple efforts to enhance the activity of long-course chemoradiation with intensified concurrent regimens [5,6,7,8,9,10].

Advancing all planned chemotherapy promptly into the preoperative setting might increase compliance, expose micro-metastatic disease, and provide additive antitumor activity—the rationale for TNT strategies. On the other hand, TNT represents a kind of “short-cut” in the treatment decision-making for LARC, as delivery of chemotherapy pre-operatively avoids the dilemma of selecting patients for postoperative adjuvant chemotherapy based on baseline MRI high risk features or post-treatment histopathology findings. It is an easy decision, but it limits the prospect of personalized treatment for LARC. Despite this, indiscriminate chemotherapy intensification for all patients, i.e., TNT, is more acceptable in the oncologic community compared to post-operative adjuvant chemotherapy.

Three phase III trials employing TNT in patients with LARC have been published recently: RAPIDO (Rectal Cancer and Preoperative Induction Therapy Followed by Dedicated Operation), PRODIGE-23 (Partenariat de Recherche en Oncologie Digestive), and STELLAR (Short-Term Radiotherapy Plus Chemotherapy Versus Long-Term Chemoradiotherapy in Locally Advanced Rectal Cancer) [11,12,13,14,15,16,17]. In addition, two sizeable randomized phase II trials compared the use of doublet chemotherapy either as induction prior to LCCRT or consolidation following LCCRT [18,19].

Current NCCN guidelines recommend neoadjuvant therapy for all patients with clinical T3, cNany with clear CRM (defined by MRI), and cT1-2, cN1-2 (NCCN 2022). TNT with a 12- to 16-week duration of FOLFOX or CAPEOX is preferred as the standard for all more advanced clinical stages [20]. There is no longer any distinction between lower/mid and upper rectal cancers. Current ESMO guidelines predate recent TNT results [1].

The approach of delivering TNT to all patients with rectal cancer as standard of care does not square with our knowledge of its inherent clinical, genetic, and immunological heterogeneity and the goal of personalized medicine. In addition, only a small proportion of patients actually benefit from RT or chemotherapy (particularly in stage II). We also recognize a substantial risk of clinical overstaging with current imaging and the few poorly effective salvage treatments in CRC after poor response or relapse. Nor does this blanket strategy recognize the potentially unnecessary acute and late toxicity experienced by many patients.

Yet, we lack the ability to be selective, as there are no clinically practical predictive factors for the effectiveness of chemotherapy and radiotherapy. Additionally, convincing risk factors to individualize patients more likely to benefit from TNT are yet to be identified, and the impact of TNT on distant metastases appears similar in all subgroups. These observations, coupled with the enhanced opportunity for NOM, explain why TNT is a popular treatment option.

The aim of this descriptive review was to examine the current evidence for the routine use of TNT for patients with LARC, to clarify the relevant evidence, and to weigh the quality of that evidence both for small early-stage tumors and more advanced ones with more aggressive features. Based on the evidence from the randomized trials, we intended to determine if any particular group of patients is more appropriate for the use of TNT than others. We examined if there were any necessary or sufficient criteria for the use of TNT. Finally, we hoped to clarify if any particular aims of treatment were more likely to be achieved by the use of a particular schedule of TNT.

## 2. Methods

### Search Strategy and Selection Criteria

References were retrieved from two electronic databases (PubMed and MEDLINE), which were searched for published articles and abstracts from international meetings containing data from retrospective, prospective, and randomized clinical trials investigating total neoadjuvant therapy. We also searched ClinicalTrials.gov and ISRCTN. We extracted data, reviewed the list of retrieved articles, and selected potentially relevant articles. We categorized TNT as including a minimum of at least 12 weeks of FOLFOX/CAPEOX/FOLFIRINOX in the experimental arm and followed intention-to-treat analysis for the endpoints.

We found six relevant phase III trials exploring LCCRT or SCPRT with the addition of neoadjuvant induction or consolidation doublet or triplet chemotherapy, i.e., NSABP-R03, Polish 2, FOWARC, RAPID0, STELLAR, and PRODIGE-23 [11,12,13,14,15,16,17,21,22,23,24,25]. Randomized phase III trials examining the addition of oxaliplatin concurrently with LCCRT provided useful data for benchmarking [5,6,7,8,9,10,26,27]. We also considered several relevant randomized phase II trials investigating NACT [18,19,28,29,30,31,32,33,34,35,36,37,38,39,40], prospective phase II trials, and meta-analyses of varying quality examining TNT [41,42,43,44,45,46,47,48,49,50].

## 3. Results

### 3.1. Available Evidence/Founding Trials

In the single-agent fluoropyrimidine era, the NSABP R-03 phase III trial pioneered induction NACT with an experimental arm delivering six weeks of 5FU/FA followed by LCCRT. A short-duration NACT (6 weeks) before preoperative LCCRT was feasible with a significant improvement in 5-year DFS compared with postoperative LCCRT—64.7% vs. 53.4%, respectively (*p* = 0.011), but no benefit in OS [21]. This short NACT schedule was not widely adopted—only the preoperative LCCRT component was considered a confirmatory study for the previous German landmark trial [2].

Other studies investigating the addition of more intensive NACT to LCCRT using fluoropyrimidine/oxaliplatin doublets illustrated improved compliance and less toxicity compared to LCCRT and postoperative adjuvant chemotherapy. Results did not show improvements in pCR, DFS, or OS [28,29], but an exploratory analysis identified good compliance with induction chemotherapy (i.e., 3–4 cycles received) as a factor associated with a lower risk of recurrence [29].

The Polish 2 phase III trial in borderline/unresectable cancers compared SCPRT followed by 3 cycles of consolidation FOLFOX and surgery against LCCRT and surgery. Initial results reported improved OS [22], leading to the schedule’s incorporation in ESMO guidelines [1]. This benefit in OS disappeared with a longer follow-up [23].

In a retrospective analysis from MSKCC, the rate of sustained clinical complete response (CCR) was almost doubled in patients receiving NACT compared to those treated with LCCRT and post-operative adjuvant chemotherapy [51]. This study formed the rationale for the subsequent randomized phase II “OPRA” trial [19].

Both the recent RAPIDO and PRODIGE-23 phase III trials reported a doubling of the pCR rate with TNT compared with standard LCCRT alone. There was also a statistically significant improvement in disease-related treatment failure (DRTF) [12] and DFS [14], with a significant reduction in the rate of metastases [12,13,14] compared with standard LCCRT. The Chinese phase III trial (STELLAR) with a similar design to RAPIDO reported a similarly high combined pCR and CCR rate of 24.2% [16], further supporting the view that TNT increases options for organ preservation. This latter study did not report improvement in DFS or distant metastases control but, surprisingly, a significantly better OS. The characteristics (Table 1) and outcomes (Table 2) of these trials are summarized in the tables. Other data appears to confirm that TNT, and particularly consolidation CT, can enhance CCR rates, thereby facilitating options to explore organ preservation [19,52]. These results have led to calls for TNT to be adopted as a standard of care in guidelines.

### 3.2. Eligibility Criteria

Characteristics included as eligibility criteria for the phase III trials vary widely. RAPIDO used MRI to select for highly adverse features, i.e., cT4, cN2, EMVI, threatened CRM, and enlarged LPLN [12]. Stratification was according to center, performance status, cT stage (cT2–cT3 or cT4), and cN stage (cN− or cN+).

PRODIGE-23 enrolled patients with cT3 (at risk of local recurrence) or cT4. They mandated ultrasound for patients with cT3N0 to exclude cT2 tumors. Yet they enrolled 43% of the population with cT2/cT3a/cT3b in the novel arm, and likely a proportion of these were stage II. Stratification was according to center, depth of extramural extension (≥5 mm vs. <5 mm), tumor location, and stage (cT3 vs. cT4; cN0 vs. cN+) [4]. In STELLAR, also with broader inclusion criteria—i.e., cT3, cT4, cNany—they recruited 159/302 (52.5%) of patients with cT2/cT3a/cT3b in the novel arm [16] with stratification by location, clinical stage, and MRF status. Rectal cancer has a peak incidence at age 80, but the average age of participants in clinical trials is younger. Both the RAPIDO and PRODIGE-23 trials recruited patients with limited co-morbidity, a median age of 62 years, and 90% and 80%, respectively, had PS = 0 [12,14]. In the Stellar trial, the median age was even younger, 55/56 years, and PS = 0 in 85% [16]. In contrast, population data suggest that 70% of patients presenting with rectal cancer are aged >65 years [53].

### 3.3. Toxicity

Acute detailed toxicity from the randomized trials is reasonably accurate, but patient-reported outcomes (PROs) report higher rates of toxicity than clinician-reported studies, which include a narrower scope and limit to more severe (grade ≥ 3) symptoms [54].

In the PRODIGE-23 trial, grade 3–4 adverse events occurred in 105 (46%) of 226 patients in the neoadjuvant chemotherapy group [14]. G-CSF, prescribed on a case-by-case basis, was administered to 61/226 (27%) of patients. In RAPIDO, rates of grade 3/4 adverse events were reported in 48% of cases during TNT for the experimental arm, compared with 25% in the standard arm, but 35% also reported grade ≥ 3 toxicity with post-operative adjuvant chemotherapy [11]. Diarrhea was the commonest grade ≥ 3 toxicity in the neoadjuvant setting (18% experimental vs. 9% standard arm). A more detailed study on quality of life and late toxicity [15] has been published. In STELLAR, TNT was associated with an almost doubling of grade ≥ 3 toxicity compared with standard LCCRT (26.5% vs. 12.6%) (*p* = 0.001) [16]. Compliance and toxicity for these studies are tabulated in Table 3.

### 3.4. Early Endpoints of Response

#### pCR/CCR

Achievement of an excellent response to LCCRT or TNT—i.e., CCR or a pCR—is both associated with a favorable prognosis, and CCR will facilitate options such as the “watch and wait” strategy—avoiding radical surgery. The trial protocol has to be considered in evaluating CCR and pCR rates. Both PRODIGE-23 and RAPIDO mandated TME surgery after completion of neoadjuvant treatment, and a watch-and-wait strategy was considered a protocol violation. Thus, few patients underwent watch and wait after achieving a CCR (2 and 14 patients, respectively, in PRODIGE-23 and RAPIDO) (Table 4) [9,10]. These two trials reported a doubling of the pCR rate compared with standard LCCRT. In contrast, in STELLAR, NOM was permitted and pCR was reported in 39/235 (16.6%) and CCR with NOM in 28/298 (9.4%), 2 of whom had subsequent regrowth [16].

### 3.5. Resection Margins

The status of the distal and circumferential margins is a high-risk factor determining local and distant recurrence following surgery [55,56]. The distance of the tumor from the anal verge and the need for abdominoperineal resection are potential hazards for a subsequent involved circumferential resection margin (CRM) and usually direct selection of LCCRT to achieve downstaging. One of the arguments proposed for TNT is that additional chemotherapy enhances response and is likely to improve R0 resection rates. However, in both RAPIDO and PRODIGE-23, R0 resection rates in the TNT arm and the control arm were almost identical: 82/423 (90%) versus 360/398 (90%) in the control arm in RAPIDO, and 95% vs. 94%, respectively, in PRODIFE-23. The relevant rates in the Stellar trial were 215/235 (91.5%) vs. 202/230 (87.8%) in the control LCCRT arm (Table 2). Phase III trials provide no evidence that TNT increases the rate of negative resection margins compared to standard LCCRT. A recent minireview of TNT confirmed this finding in a wider scope within 6 RCTs, which were available for 2268 patients. The authors summarized that R0 was achieved in 1102/1225 patients (90%) in the TNT experimental arm and 959/1043 patients (92%) in the control arm, i.e., showing comparable R0 resection rates [49].

### 3.6. Long-Term Oncological Outcomes

The main phase III trials of TNT include RAPIDO, STELLAR, and PRODIGE-23 [12,14,16]. All started with the same primary endpoint—survival (DFS) at 3 years. However, the RAPIDO investigators switched the primary endpoint in 2016 (during the trial) to DRTF at 3 years, defined in the publication as “the first occurrence of locoregional failure, distant metastasis, new primary colorectal tumor, or treatment-related death, assessed in the intention-to-treat population”. Curiously, for a chemotherapy intensification trial, this definition excludes non-cancer deaths and non-colonic second malignancies.

RAPIDO and PRODIGE-23 reported their primary endpoints met with a statistically significant decrease in DRTF in RAPIDO from 30.4% to 23.7% (*p*: 0.019). PRODIGE-23 also reported a primary endpoint met with a statistically significant increase in DFS from 69% to 76% (*p*: 0.034) (Table 1). Both trials reported a reduction in the rate of distant metastases of 7% in PRODIGE-23 and 6.8% in RAPIDO compared to the rate in the standard LCCRT arm. Hence, there was an identical level of benefit in their primary endpoints in both trials. Initial results showed similar levels of locoregional control (Table 1), suggesting the effect of TNT is predominantly preventing metastases.

With longer follow-up, in the RAPIDO trial, locoregional recurrence (LRR) is significantly higher (44/431 (10%) vs. 26/428 (6%); *p* = 0.027) and more frequent in cases with a breached mesorectum (9/44 (21%) vs. 1/26 (4%); *p* = 0.048 [17]. The explanation for this finding remains unclear, but this increase in LRR reduces the significance of the primary endpoint (DRTF), and the present HR (0.79) is less than originally reported.

Both PRODIGE-23 and RAPIDO trials showed similar 3-year overall survival (OS) in both arms: 89.1% (95% CI 86.3–92.0) in the experimental TNT arm of RAPIDO versus 88.8% (95% CI 85.9–91.7) with standard LCCRT (*p* = 0.59), and 91% (95% CI 86–94) with FOLFIRINOX in the TNT arm versus 88% (95% CI 83–91) in the standard LCCRT in PRODIGE 23 [12,14].

In contrast, however, in STELLAR, the ITT local recurrence was 20/302 (6.6%) with TNT versus 23/297 (7.7%) in the LCCRT arm. Patients who achieved an R0 resection with a CRM > 1 mm had a recurrence rate of 8/215 (3.7%) in the TNT arm versus 13/202 (6.4%) in the control arm. (Table 1). The median duration of follow-up was only 35.0 (range, 8.3–63.9) months. The 3-year DFS is similar in both arms—64.5% vs. 62.3%—but the design delivered 3–6 months of chemotherapy in both arms, a potentially relevant difference compared to RAPIDO. Despite this lack of improvement, the study was considered positive because of the non-inferiority design. Of note, the 3-year DFS in the LCCRT control arm (62.3%) was lower than previous LARC cancer trials. Many patients failed to undergo surgery (20% of the total study population, 63 patients in both arms). In total, 36/293 (12.2%) with tumor present refused surgery, and 14/293 (4.8%) progressed prior to surgery in the standard LCCRT arm, compared with only 17/298 (5.7%) and 11/298 (3.7%) in the experimental arm, respectively, a 7.6% difference [16]. This deficiency makes the results difficult to interpret—in particular, the significant difference in OS at 3 years, 86.5% in the experimental arm versus 75.1%—HR = 0.67 (95% CI, 0.46 to 0.97), *p* = 0.033.

At variance with RAPIDO, in STELLAR, acute toxicity was increased compared to the standard arm. Thus, the imprecision in surgery planning, the increased toxicity, and the absence of a benefit in disease control (DFS, DM, and LR) do not allow STELLAR to be considered a confirmatory trial of RAPIDO results [57].

The published median follow-up of 46.5 months in PRODIGE-23 and 56 months in RAPIDO [12,14] (with an update to 64 months) [13,17], makes it unlikely that a clinically meaningful difference in OS will ever be observed.

### 3.7. Quality of Life (QOL)

Based on patient-reported outcomes (PROMs), many patients experience clinically significant symptoms during chemotherapy and pelvic LCCRT. Diarrhea and urgency are commonly reported, but these symptoms are often underestimated on clinician-reported assessments [58]. In the setting of TNT, induction chemotherapy may assist compliance, as it is associated with lower odds of experiencing urgency, bleeding, and tenesmus on PROs during subsequent LCCRT, although subsequent dose modifications may also have been helpful. There was no significant impact on diarrhea or rectal pain [59].

QOL in PRODIGE-23 was assessed using the generic European Organization for Research and Treatment of Cancer Quality of Life Questionnaire Core-30 (EORTC QLQ-C30) and the short, specific colorectal cancer module (QLQ-CR29). Questionnaires were completed at baseline and for 3 years or until progression [14]. Global health status and HRQOL scores started low but improved over time in both groups (*p* < 0.0001). A detailed comparison of QOL results is promised in a future publication.

RAPIDO examined health-related quality of life (HRQL), bowel function, and late toxicity only in patients without DRTF at 3 years. The authors used the EORTC QLQ-C30 and QLQ-CR29 (with additional questions related to sexual functioning and the assessment of chemotherapy-induced peripheral neuropathy). They also reported the incidence of low anterior resection syndrome (LARS) [15]. The authors found no significant differences in HRQL, bowel function, or late toxicity between the patients receiving TNT or standard LCCRT (+/− postop adjuvant chemotherapy). Yet it should be noted that three years after surgery, the majority of patients—59% in the experimental arm and 75% in the standard LCCRT group—experienced major LARS. It should also be noted that one third of patients in the RAPIDO trial had tumors in the upper third, >10 cm from the anal verge, some of whom would have had a partial mesorectal excision (PME) and a colorectal anastomosis, which makes the overall incidence of major LARS even more concerning. Thus, a major price is paid in terms of compromised late functions for both groups. In the STELLAR trial, no data on QOL are yet available [16].

In summary, intensified treatment with TNT does not compromise QOL compared to standard LCCRT, but equally, the positive oncological outcomes deriving from TNT do not lead to better QOL.

### 3.8. Non-Operative Management and Sphincter Sparing Opportunities

Most patients express a strong preference to avoid a major operation, particularly an abdominoperineal resection (APER), which is associated with significant morbidity, a permanent stoma, and substantial quality of life effects [60]. TNT is used as much in the hope of achieving more organ sparing and avoiding such surgical sequelae in a wider group of patients as it is in the hope of improving long-term oncological outcomes in a substantial proportion of cases.

As described above, the RAPIDO and PRODIGE-23 trials mandated surgery and discouraged NOM. The use of TNT, despite clear evidence of increased pCR and down-staging from intensification, did not result in less radical operations or sphincter sparing in any of the three trials.

In PRODIGE-23, similar proportions of patients underwent low anterior resection (168/213 (78.9%) in the TNT arm versus 160/215 (74.4%) in the control arm); abdominoperineal resection (14.1% versus 14.0%); and inter-sphincteric resection (7.0% versus 10.7%) [14]. In RAPIDO, anterior resection was performed in the TNT arm in 248/426 (58%) versus 223/400 (55%) in the control arm; abdominoperineal resection in 149/426 (35%) versus 160/400 (40%); and Hartmann’s procedure (4.7%) versus (3%). These differences are not easily explained because the proportion of tumors in the low rectum < 5 cm from the anal verge was lower in RAPIDO (22%), compared with 38% and 48% in PRODIGE-23 and STELLAR, respectively, and 32% of tumors in RAPIDO were sited in the upper rectum >10 cm from the anal verge. The types of surgery in the STELLAR trial included abdominoperineal resection in 106/235 (45%) versus 95/230 (41%), anterior resection in 111/235 (47%) versus 121/230 (52%), Hartmann procedure in 13 (5.5%) versus 8 (3.5%), and others in 5 (2.1%) versus 6 patients (2.6%), in the TNT arm and control arm, respectively [16]. Hence, the avoidance of a permanent stoma is not enhanced by a TNT strategy.

### 3.9. Selection of Patients Deriving the Most Benefit from TNT

Ideally, the benefit of TNT in reducing metastatic disease needs to be targeted by identifying patients who will benefit from this additional treatment. Modern imaging with MRI can identify features recognized as independent poor prognostic indicators (i.e., cT4, threatened or involved CRM, EMVI, tumor deposits, and lateral pelvic lymph node involvement), which impact both local recurrence and the subsequent risk of metastatic disease. Yet, there is no indication from the Forest plots of patient characteristics in these phase III trials to suggest that patients with any particular adverse features experience any additional statistically significant benefit from TNT in terms of DFS over and above any benefit gleaned from LCCRT alone. The only features that do not cross unity are younger age, good PS, early cT stage, no EMVI, and low CEA levels—but include cN+ [12,14].

## 4. Discussion

### 4.1. TNT as Standard Treatment-Pros and Cons

All three phase III studies testing TNT achieved their primary endpoint. RAPIDO and PRODIGE 23 show a doubling of the pCR rate with TNT and a significant improvement in DFS or DRTF—mainly reflecting a 7% reduction in the incidence of metastases, but no improvement in OS. Previous phase III trials intensifying pre-operative chemoradiation regimens and investigating adjuvant chemotherapy were broadly negative. This benefit of reducing distant metastases is unprecedented in LARC and provides motivation for wider use of this strategy. It is curious that in RAPIDO, intensification with additional neoadjuvant chemotherapy increased the pCR and CCR rates and reduced the metastatic rate but had no effect on local control. Although unexplained, the significant increase in local recurrence will temper the use of this schedule in the future for patients considered at high risk of local recurrence. Yet in earlier cancers, TNT may increase cCR and therefore facilitate NOM. The latter results make TNT attractive as a preferred option for many medical oncologists, radiation oncologists, and surgeons.

All the phase III trials supporting TNT as a standard treatment have major biases. In RAPIDO, the increase in pCR rates could be partly attributed to the longer interval from the end of RT to surgery compared to the standard arm (23 weeks). Similarly, in PRODIGE 23, a contribution from Irinotecan cannot be excluded. Previous data from Polish II with an 11-week interval before surgery showed only a marginal but not significant improvement in pCR.

The optional adjuvant chemotherapy policy in the control arm of RAPIDO also flaws the interpretation of the decreased metastasis rate and limits general conclusions. In STELLAR, where full, protocol-prescribed, oxaliplatin-based adjuvant chemotherapy was included in the comparator standard arm, there was no improvement in DFS. In PRODIGE-23, the experimental arm includes both induction chemotherapy and intensification with the addition of irinotecan, as well as 3 months of postoperative adjuvant chemotherapy. In STELLAR, the large proportion of patients not undergoing surgery is a major bias. The main strength of RAPIDO and PRODIGE 23 is thus the inter-study concordance of the results rather than internal robustness.

Unanswered questions remain. First, none of these studies clearly proves that the tested TNT regimen, per se, determines improved distant control. With induction NACT, it is unclear whether the reduction of distant metastases observed in PRODIGE-23 is related to up-front and optimized delivery of chemotherapy or the use of a triplet chemotherapy regimen (FOLFIRINOX). Many have extrapolated from the results of RAPIDO to deliver up-front chemoradiation followed by consolidation with FOLFOX/CAPEOX. Doublet regimens (FOLFOX or CAPEOX) either as induction or consolidation in combination with LCCRT have not been shown to be superior to LCCRT in any phase III trials, but oncologists feel comfortable with these schedules.

Second, routine TNT use would imply a less selective approach towards the use of systemic chemotherapy in LARC, resulting in potential overtreatment in some groups of patients. In contrast, induction chemotherapy might modulate the need for radiotherapy in some groups of patients, which would be helpful to further justify the costs of TNT.

Third, TNT does not appear to have any effect on local control. LCCRT probably continues to be necessary for high-risk patients.

The variable eligibility criteria in these trials and the different oncological approaches create challenges in selecting the optimal strategy to achieve local control and cure while minimizing morbidity for each individual patient. This difficulty is compounded by the fact that the Forest plots do not identify any adverse features, which clearly benefit, or low-risk features, which do not benefit from TNT in terms of improved DFS.

Current ESMO guidelines do not routinely recommend LCCRT or SCPRT for locally advanced upper rectal cancer [1], reflecting the low local recurrence rates of upper rectal cancer in the MRC-CR7/NCIC-CTG-C016 and the CAO/ARO/AIO-94 trials (4.7% and 2.5%, respectively). ESMO clinical practice guidelines utilize the ability of high-quality MRI to subclassify cT3, which is recommended. ESMO guidelines state that neoadjuvant radiotherapy may be omitted for patients with MRI-defined cT3a/b and cN0–1 in the middle and upper-third of the rectum because these patients have a low risk of local recurrence. These stark disparities imply that either ESMO guidelines recommend substantial undertreatment or NCCN guidelines recommend substantial overtreatment for patients with rectal cancer.

There are no available clinical, molecular, immunological, or imaging features that either direct or aid these choices. Nor does this strategy always balance the loss of fertility, acute toxicity, late sexual, urinary, and bowel problems, in addition to the time spent and substantial financial toxicity suffered by patients from TNT.

### 4.2. Induction vs. Consolidation

There is considerable diversity with different potential schedules and a multitude of approaches with possible combinations. The different sequences of induction and consolidation chemotherapy around a LCCRT platform have advantages and disadvantages. There is no consensus as to the role and optimal positioning of NACT in a TNT schedule. These options include induction neoadjuvant chemotherapy (INCT) prior to LCCRT with several chemotherapy options (CAPEOX, FOLFOX, and FOLFOXIRI) lasting 6–18 weeks or consolidation neoadjuvant chemotherapy (CNCT) following SCPRT or LCCRT, respectively. However, we are far from defining the optimal/ideal combination or sequence of a TNT approach.

When directly compared, the OPRA trial showed better chemotherapy compliance with induction chemotherapy over consolidation chemotherapy (99% vs. 94%), but radiation compliance was inferior (93% vs. 98%) [19]. These results are consistent with the results of CAO/ARO/AIO-12 [18]. However, DFS was no different in both trials [18,19].

### 4.3. Overtreatment/Unnecessary Treatment

Patients with resistant cancers experience unnecessary side effects if treatment continues for no oncological gain. They may be further disadvantaged by a delay in switching to other potentially more effective treatment strategies (if available) or a less prompt and suitable exit strategy, such as surgery. Hence, a consolidation-CT TNT strategy seems more appropriate for patients with an observed good response following neoadjuvant LCCRT, which can be improved further, rather than a non-responding tumor. Thus, accurate early response assessment is crucial if adverse outcomes are to be avoided [61]. Alternatively, a biomarker that predicts response or non-response to LCCRT or chemotherapy at an early time point is the “holy grail” in rectal cancer.

Induction chemotherapy offers an early opportunity to identify patients with a more aggressive/chemo-resistant biology. One of our major concerns regarding TNT administration is related to the minimal effect of TNT on larger and more advanced cancers, which do not appear to undergo similar down-staging as smaller, early-stage tumors. Such patients are unlikely to benefit from standard fluoropyrimidine based LCCRT—although waterfall plots confirmed some tumor shrinkage with LCCRT following stable disease after FOLFOX [62]. The data does not inform whether this shrinkage was clinically relevant and sufficient to attain an R0.

Yet apart from the option of standard-dose radiation or dose escalation for poor responders, we currently lack any different, more intensified regimens with proven efficacy. Hence, after induction of NACT, we need an effective exit or “off ramp” strategy for poor responders beyond automatically proceeding to standard fluoropyrimidine-based chemoradiotherapy or salvage surgery.

The use of FOLFIRINOX as in PRODIGE-23 may be different. The results of the small GRECCAR 4 randomized phase II trial (*n* = 206) support the feasibility of using a graded response to FOLFIRINOX induction chemotherapy to select different options to reinforce or salvage good and poor responders, respectively. Yet the initial response still determined long-term outcomes [63]. The strategy of dose-escalation is feasible, but it does not appear to increase R0 resection rates or provide advantages in terms of 5 year local control, DFS, or OS [63]. However, there is no data to confirm the same results can be achieved with FOLFOX alone.

In addition, results are paradoxical in that none of the phase III trials appear to show that the perceived assessment of respectability was enhanced after TNT. The R0 resection rate did not increase, and the quality of the TME specimen was in fact non-significantly worse in the RAPIDO and PRODIGE trials (as assessed by the surgeon and pathologist, respectively). These findings do not imply, according to the present authors, that TNT makes surgery less complex or more likely to be curative.

### 4.4. Duration of TNT

TNT has no universally agreed-upon optimal schedule in terms of radiotherapy, chemotherapy agents, or duration. Large, randomized studies in stage III and high-risk stage II colon cancer showed that a shorter duration of postoperative adjuvant CAPOX (3 months) provides virtually equivalent overall survival (OS) and DFS as the historical 6-month comparator [64]. Three months of CAPOX reduced toxicity and costs. Yet the evidence base supports either 3 months of induction with FOLFOXIRI or 18 weeks of consolidation with CAPOX.

### 4.5. Future Developments

Current NACT options, even when combined with SCPRT or LCCRT, suggest a ceiling effect in terms of response in LARC, so novel alternative intensification treatment strategies are needed. Dose-escalation of radiotherapy, although enhancing opportunities for NOM in small early tumors (OPERA), does not seem to have been so effective in high-risk patients. Therefore, novel strategies are required, which may be tailored to the molecular profile or provided by immunotherapy. Liquid biopsy integrating circulating tumor DNA (ctDNA) may give an early readout of effectiveness and drive both escalation and de-escalation strategies.

De-intensification strategies are also being discussed. Some have advocated the use of NACT without chemotherapy in selected patients. The PROSPECT trial rationale relies on the argument that if predicted lateral or distal margins are not threatened on MRI staging and an APER (which carries a high risk of a positive CRM) does not need to be performed (i.e., an upper or mid rectal cancer), then the risk of distant metastases far outweighs the risk of local recurrence. Thus, chemotherapy alone should be able to counter potential distant micrometastases, provided a clinical response is observed, will be sufficiently effective to prevent local recurrence. The eligibility criteria for PROSPECT overlap to a large degree with low-risk categories, from which current ESMO guidelines allow LCCRT to be omitted.

A French randomized phase III trial (NORAD01) (NCT03875781) is running in patients with easily respectable LARC (cT3N0 or cT1-T3N+ with CRM  >  2 mm), which randomizes patients between modified FOLFIRINOX alone (as used in PRODIGE-23) for 3 months and standard LCCRT using the primary endpoint of 3-year progression-free survival (PFS). It is a non-inferiority trial aiming to assess if equivalent oncological outcomes are associated with less late toxicity after FOLFIRINOX.

There are two interesting German phase III trials. ACO/ARO/AIO-18.1 (NCT04246684) is a German phase III trial designed to define the optimal RT platform in MRI-defined intermediate and high-risk patients. A total of 702 patients are to be randomized between a RAPIDO-like schedule of 5 × 5 Gy followed by 18 weeks of CAPEOX or FOLFOX consolidation and the German concurrent LCCRT platform of fluoropyrimidine/oxaliplatin followed by the same duration of FOLFOX/CAPEOX with a primary endpoint of organ preservation at 3 years.

This trial complements the study ACO/ARO/AIO-18.2 (NCT04495088) in low-risk patients, which randomizes 818 participants between 3 months of preoperative FOLFOX or CAPEOX chemotherapy and immediate surgery followed by stage- (risk-)adapted adjuvant chemotherapy.

GRECCAR 14 (NCT04749108) has an adaptive design that selects treatment according to early primary tumor response to induction FOLFIRINOX. A total of 430 patients are to be enrolled, and “very good” responders (according to MRI-defined volumetric tumor response) are randomized between immediate surgery and LCCRT followed by surgery. Primary endpoints are the R0 resection rate (CRM > 1 mm) and 3-year DFS.

There are also ongoing Chinese phase III trials, i.e., “Total Neoadjuvant Treatment vs. Chemoradiotherapy in Local Advanced Rectal Cancer With High Risk Factors (TNTCRT)” (NCT03177382, 458 participants), and a sandwich design “Randomized Controlled Study on Optimize Neoadjuvant Chemoradiotherapy for Locally Advanced Rectal Cancer” (NCT02031939) with 556 participants due publication within the next 4 years.

## 5. Conclusions

TNT delivers earlier systemic therapy with adequate doses and better compliance than postoperative adjuvant chemotherapy, although the optimum sequence, duration, radiotherapy platform, and schedule are undefined. TNT is safe and does not significantly compromise subsequent LCCRT or surgery, with minimal risk of tumor progression at 3 months.

TNT enhances response, and more patients achieve a pCR/CCR, which facilitates NOM for a larger proportion of patients, but the risk of regrowth remains and may be higher with induction chemotherapy compared to consolidation. Given these gains from TNT, QOL is not impaired, but the duration is prolonged and may have financial consequences for patients.

TNT reduces the risk of metastases in 7% of cases, but currently there are no gains in OS. As a rule, clinicians require both a significant benefit in DFS or DRTF and an improvement in OS before considering that the additional toxicity from TNT is sufficient to adopt a regimen as a standard of care.

The cost of TNT to healthcare is increased only in so far as compliance with neoadjuvant chemotherapy is considerably better than postoperative chemotherapy, and NOM, if achieved, is cost-saving.

However, with the exception of the RAPIDO trial, patients with prognostically unfavorable tumors are underrepresented in the phase III trials. This omission hampers our ability to select high-risk patients who are most suitable for TNT on an individual basis. Yet the overall reduction in metastases observed in a low- to middle-risk population supports the role of preoperative chemotherapy and suggests a potentially larger use of this strategy.

Yet, the data from the phase III trials is insufficient to allow a decision between LCCRT and SCPRT as the best option for the radiotherapy platform. In the future, the answer may be clarified in part by the results of the AIO-18.1 trial. It is also unproven how much additional benefit neoadjuvant FOLFIRINOX offers over and above the doublet FOLFOX or CAPOX. The authors offer their own personal views as to their recommendations for the indications and use of TNT (Table 5).

For all the above reasons, predictive biomarkers are urgently required. We need to define patient characteristics where TNT can make a real difference, rather than thinking of TNT as a standard adjuvant approach suitable for all patients.

## Figures and Tables

**Table 1:** (a). Reported randomized studies of TNT/neoadjuvant chemotherapy alone in rectal cancer: baseline characteristics. (b). Reported TNT-randomized studies in rectal cancer: timing of intervals between each modality of treatment.

(a)						
	RAPIDO		PRODIGE 23		STELLAR	
No of Patients	920 Enrolled 912 Eligible		461 Randomized		599 Randomized	
	462	450	231	230	298	293
**Arm and** **regimen**	**Novel arm** **SCPRT + Capeox × 6 or FOLFOX × 9**	**Standard arm** **LCCRT +/− adjuvant**	**Novel arm** **FOLFOXIRI + LCCRT + doublet adjuvant**	**Standard arm** **LCCRT + doublet adjuvant**	**Novel arm** **SCPRT + Capeox × 4 + doublet adjuvant**	**Standard arm** **LCCRT + doublet adjuvant**
**Eligibility**	Pelvic MRI showing high-risk criteria: cT4a or cT4b, EMVI, clinical nodal (cN) stage cN2, involved MRF ≤ 1 mm from the mesorectal fascia or enlarged LPLN		cT3 (at risk of local recurrence and for which a Multidisciplinary team (MDT) board recommended preoperative chemoradiotherapy) or cT4		cT3/cT4 cN any or cN+ distal or middle third of the rectum	
**Stratification factors**	**(4)** Centre, performance status, cT stage (cT2–cT3 or cT4), and cN stage (cN− or cN+).		**(4)** centre, extramural extension (≥5 vs. <5 mm), tumour location and stage (cT3 vs. cT4; cN0 vs. cN+)		**(1)** Status of MRF (MRF+ vs. MRF−)	
**Median Age in years** **>65**	**62** 182 (39%)	**62** 188 (40%)	**62** 73 (32%)	**62** 85 (37%)	**55**	**56**
**PS = 0**	369 (80%)	365 (81%)	178/229 (78%)	182/6 (81%)	256 (86%)	250 (85%)
**cT2**	14 (3%)	14 (3%)	3/225 (1%)	2/225 (1%)	7 (2.3%)	9 (3%)
**cT3**	301 (65%)	299 (66%)	**182/225 (80.9%) *cT3a/b = 41.7%***	**188/225 (83.6%) *cT3a/b = 48.5%***	245 (82%)	248 (85%)
**cT4**	147 (32%)	137 (30%)	40/225 (18%)	39/225 (17%)	46 (15.5%)	46 (12.3%)
**cN1**	118 (26%)	120 (27%)	148 (64%)	155 (67%)	154 (51%)	147 (49.5%)
**cN2**	302 (65%)	295 (66%)	59/225 (26%)	53/225 (23%)	104 (34.9%)	99 (33.8%)
**CRM threatened**	285 (62%)	271 (60%)	48/185 (26%)	54/195 (28%)	166/298 (56%)	163/293 (56%)
**EMVI+**	48 (32%)	125 (28%)	No data	No data	159 (53%)	122 (42%)
**LPLN+**	66 (14%)	69 (15%)	23 (10%)	24 (10%)	No data	No data
**<5 cm from anal verge**	103 (22%)	115 (26%)	87 (38%)	83 (36%)	(48%)	(49%)
**>10 cm from anal verge**	**146/462 (32%)**	**151/450 (34%)**	30/231 (13%)	29/230 (13%)	2/298 (0.7)	0/293 (0)
**(b** **)**						
**Median time from start of treatment to surgery (IQR)**	24 weeks	18 weeks	184 (176–196) days	90 (84–98) days	21 weeks	24 weeks
**Median time from end of SCPRT to start of chemo**	Median 14 days	Not relevant	Not relevant	Not relevant	7–14 days	Not relevant
**Median Interval to surgery from end of chemo/LCCRT**	4 weeks	6–10 weeks	54.5 days	55 days	4–6 weeks	6–8 weeks
**Median interval to surgery from end of radiation**	8–10 weeks	22–25 weeks	7 weeks	7 weeks	9 weeks	20 weeks
**Median time from randomization to surgery**	25.5 weeks	15.9 weeks	From start of treatment 184 days (26.3 weeks)	From start of treatment 90 days (12.9 weeks)	From start of treatment 21 weeks	From start of treatment 14 weeks
**Completed neoadjuvant treatment**	389/423 (85%) completed preoperative chemotherapy	Not relevant	207/226 (92%)	Not relevant	Completed dose reduced/delayed 86.2% Completed all planned 74.8%	Completed dose reduced/delayed 95.2% Completed all planned 93.2%
**Started postoperative Adjuvant chemo**	6/423 (protocol violation)	187/398	160/207 (77.3%)	158/201 (78.6%)	235/298 (79%)	230/293 (78%)
**Completion of Postoperative Adjuvant Chemotherapy**	Not relevant	28/54 centres (52%) opted to administer postop adjuvant chemotherapy 118/187 (63%) in centres opting for chemotherapy completed postop adjuvant chemo	129/160 (80.6%)	19/158 (75.3%)	141/235 (60%)	111/230 (48%)

**Table 2:** Long-term outcomes and short-term early endpoints.

	RAPIDO		PRODIGE 23		STELLAR	
No of Patients	920 Enrolled 912 Eligible		461 Randomized		599 Randomized (Non-Inferiority)	
	462	450	231	300	300	293
	**Novel arm** **SCPRT + Capeox × 6**	**Standard LCCRT/+/− adjuvant**	**Novel arm** **FOLFOXIRI + LCCRT**	**Standard LCCRT + adj**	**Novel arm** **SCPRT + Capeox × 4**	**Standard LCCRT+ adj**
**Underwent surgery**	**423**	**398**	**213**	**215**	**235**	**230**
**Primary endpoint**	3 year DRTF 30.4% vs. 23.7% HR 0.75, *p* = 0.019		3 year DFS 75.5% vs. 68.5%) HR 0.69, *p* = 0.03		3 year DFS (non inferiority) 64.5 vs. 62.3% HR 0.88 *p* ≤ 0.001	
**pCR rate resected** **and ITT**	120/423 (28.4%) ITT 26%	14.3% *p* ≤ 0.0001 ITT 12.6%	59/212 (27.8%) ITT 25.5%	26/215 (12%) *p* ≤ 0.0001 ITT 11.3%	39/235 (16.6%) ITT 13%	27/230 (11.8%) NS ITT 9.2%
**Median NAR score**	No data	No data	8.4	15.0	No data	No data
**Mean NAR score**	No data	No data	11.2	16.1	No data	No data
**R0 resection rate**	90%	90%	95%	94%	91.5%	87.8%
**ypT2**	82/423 (19%)	96/398 (24%)	57/212 (27%)	62/215 (29%)	73 (31.1%)	64 (27.8%)
**ypT3**	157/423 (37%)	190/398 (48%)	77/212 (36%)	103/215 (48%)	106 (45.1%)	113 (49.1%)
**ypT4**	36/423 (9%)	25/398 (6%)	4/212 (2%)	4/215 (2%)	7 (3.0%)	10 (4.3%)
**ypN+**	106/423 (25%)	125/398 (31.4%)	37/212 (17.6%)	69/215 (32%)	67 (28.4%)	70 (30.6%)
**Acute G3/4 toxicity**	48% during NACT	25% during LCCRT	47% during NACT	36% during LCCRT	26.5% during NACT	12.6% during LCCRT *p* < 0.001
**Treatment related death**	3%	3%	4%	5%	No data? 1/298 (0.3%)	No data? 1/293 (0.3%)
**Sphincter sparing procedures**	58.9%	56.2%	85.9%	85.1%	47.2%	52.6%
**APER rate**	149/426 (35%)	160/400 (40%)	30/213 (14.1%)	30/215 (14.0%)	106/235 (45.1%)	95/230 (41.3%)
**3-year Locoregional failure**	8.3%	6% NS	4.8%	5.6% NS	8.5%	11.1% NS
**3-year survival without mets**	78.8%	71.7%	80%	73.2%	76.8%	75%
**3-year overall survival**	89%	89%	91%	88%	86.5%	75.1% HR = 0.67 *p* = 0.038

**Table 3:** Randomized phase II trials comparing the sequence of induction and LCCRt vs. LCCRT and consolidation chemotherapy.

	Induction Chemotherapy + LCCRT	LCCRT + Consolidation Chemo	Level of Significance
OPRA			
**Number of pts**	158	166	
**Chemotherapy**	8 cycles of FOLFOX/6 cycles of CAPEOX	8 cycles of FOLFOX/6 cycles of CAPEOX	
**LCCRT**	5FU/cape + 50.4–54 Gy (median 54 Gy)	5FU/cape + 50.4–56 Gy (median 54 Gy)	
**DFS**	76%	76%	NS
**Metastasis free survival**	84%	82%	0.83
**TME free survival**	41%	53%	0.016
**Local recurrence free survival**	94%	94%	
**% regrowth after NOM**	42/105 (40%)	33/120 (27.5%)	
**% local recurrence**	10/158 (6.6%)	15/166 (9%)	
**CAO/ARO/AIO-12**			
**Number of pts**	156	150	
**Chemotherapy**	3 cycles of FOLFOX iNACT only	3 cycles of FOLFOX cNACT only	
**LCCRT**	5FU/oxaliplatin + 50.4 Gy	5FU/oxaliplatin + 50.4 Gy	
**pCR**	17%	25%	
**Combined PCR and CCR**	21%	28%	
**3-year cumulative incidence of locoregional recurrence**	6%	5%	0.67
**3-year DFS**	73%	73%	0.82
**3-year cumulative incidence of distant metastases**	18%	16%	0.52

**Table 4:** (a) Trials of TNT—examining randomized phase II and phase III trials—focusing on the arm delivering at least 6 weeks of consolidation chemotherapy as a component of TNT to determine the composite endpoint of pCR/CCR. (b) Trials of TNT—examining randomized phase II and phase III trials focusing on arms delivering at least 3 cycles of induction chemotherapy as components of TNT to determine the composite endpoint of pCR/CCR.

(a)							
Trial	No of Patients in TNT Arm	Interval from Start of Treatment to Assessment	pCR of Resected	pCR Rate (ITT)	cCR, Regardless of Watch and Wait or Surgery	cCR Rate Who Pursued NOM	pCR and CCR Rate (ITT)
**Trials mandating surgery. NOM** **discouraged or protocol violation**							
**Polish 2** SCPRT/FOLFOX × 3	261	12 weeks	37/220 (16.8%)	37/261 (14.1%)	None	None	37/261 (14.1%)
**RAPIDO** SCPRT/FOLFOX × 8	462	25.5 * weeks	120/423 (28.3%)	120/462 (26%)	14/462	14/462	134/462 (28.6%)
**AIO-12** XELOX CRT/FOLFOX × 3	150	127 days =18 weeks	38/142 (26.7%)	38/150 (25.3%)	4/150	4/150	42/150 (28%)
**Trials allowing NOM if CCR/nCCR observed**							
**STELLAR** CAPOX × 4	298	21 weeks	39/235 (16.6%)	39/298 (13%)	33/298	28/298	67/298 (22.5%)
**OPRA** LCCRT/FOLFOX × 8	166	34 weeks	Not provided? 3/33	3/166	CCR or near CCR 120/166 (72%)	CCR or near CCR 120/166 (72%)	pCR/CCR or near CCR 123/166 (74%)
**(b)**							
**Trial**	**No of patients in TNT arm**	**Interval from start of treatment to assessment**	**pCR of resected**	**pCR rate (ITT)**	**cCR rate**	**cCR rate who pursued NOM**	**pCR and CCR rate (ITT)**
**PRODIGE-23** FOLFIRINOX × 6 then LCCRT	231	184 days =26 weeks	59/212 (27.8%)	59/231 (25.5%)	2/231	2/231	61/231 (26.4%)
**GCR-3** CAPOX × 4 then LCCRT	56	19–20 weeks	8/52 (15.3%)	8/56 (14.3%)	0	0	8/56 (14.3%)
**AIO-12** FOLFOX × 3 then LCCRT	156	127 days = 18 weeks	27/142 (19%)	27/156 (17%)	Not stated	6/156 (3.8%)	33/156 (21%)
**NRG 002** TNT Control FOLFOX × 6 then 50.4 Gy + cape	95	23–28 weeks	20/68 (29.4%)	20/95 (21%)	13.6%	6/95	30/95 (31.6%)
**NRG 002** As above + Pembro arm	90	23–28 weeks	22/69 (31.9%)	22/90 (24.4%)	13.9%	1/90	23/90 (25.5%)
**Trials which encouraged CCR and** **NOM rather than surgery**							
**OPRA** FOLFOX × 8 then LCCRT (50–56 Gy)	166	34 weeks	Not provided? 3/33	3/166	CCR or near CCR 120/166 (72%)	CCR or near CCR 120/166 (72%)	pCR/CCR or near CCR 123/166 (74%)

* only the interval between randomization and surgery in the experimental group is provided; cCR—complete clinical response; pCR—pathological clinical response.

**Table 5:** Showing authors’ personal views of indications for TNT based on evidence from trials.

Scenario/Aim of Management by MDT	Strength of Indication	Indications and Rationale
If initial management plan is NOM	Strong	Consolidation preferred as less regrowth (OPRA), but triplet (FOLFIRINOX) induction possibly valuable alternative if fit and PS = 0
If initial management aim to avoid RT (preserve fertility etc.)	Moderate for low and intermediate risk rectal cancer	If predicted CRM > 2 mm, then induction doublet (or triplet in younger fit PS = 0 patients) and omit RT if good response to chemotherapy
If initial management aim to avoid/reduce metastases in the presence of high-risk features on MRI (mrEMVI G3/4, mrTD, cN2)	Weak/Moderate	RAPIDO data suggest predominantly liver metastases prevented so EMVI a concern; induction triplet (preferred) or doublet consolidation
If initial management aim to achieve shrinkage necessary to secure R0	Weak as no evidence that TNT increases chance of R0 resection in RAPIDO or PRODIGE-23	Triplet induction or doublet consolidation; if consolidation is planned early assessment and non-responding patients should undergo early surgery
If initial management aim to avoid/reduce metastases in the presence of high-risk features on MRI (mrEMVI G3/4, mrTD, cN2) or achieve R0 If mucinous/signet ring histology	Moderate	Triplet induction in younger fit PS = 0 patients and then chemoradiation as poor response to doublet chemotherapy in mucinous/signet ring histology
If MDT decisions regarding postop adjuvant chemotherapy are based on clinical stage/features (cT4 or EMVI—strong; cN(1)-2-weak) rather than pathological stage	Moderate as compliance unequivocally better with TNT	Triplet or doublet as consolidation (preferred as proven by randomized studies). Doublet-induction may be considered as compliance is anyway increased
